# Supplementary material for: An open-source framework for large-scale, flexible evaluation of biomedical text mining systems
Source: J Biomed Discov Collab. 2008 Jan 29;3:1. doi: 10.1186/1747-5333-3-1 (PMC2276192; doi:10.1186/1747-5333-3-1)
Supplement: Additional file 1 — Evaluation platform source code. The additional file contains the software evaluation framework discussed in this paper. The most current release can be downloaded from SourceForge.net [1]. See the accompanying README file for instructions on installation and use. Also included in the distribution is a collection of UIMA wrappers for some commonly used BioNLP tools and annotated corpora. [file 1747-5333-3-1-S1.zip › bionlp_uima_repository-1.0.1/docs/api/edu/uchsc/ccp/uima/annotation/package-tree.html]

edu.uchsc.ccp.uima.annotation Class Hierarchy (Center for Computational Pharmacology Util/UIMA API)


|  |  |  |
| --- | --- | --- |
| **Overview** \| **Package** \| Class \| **Tree** \| **Deprecated** \| **Index** \| **Help** | |  |
| **PREV**   **NEXT** | **FRAMES**    **NO FRAMES**     **All Classes** |  |


---


## Hierarchy For Package edu.uchsc.ccp.uima.annotation

**Package Hierarchies:**: All Packages

---

## Class Hierarchy

- java.lang.**Object**
  - org.apache.uima.cas.impl.FeatureStructureImpl (implements java.lang.Cloneable, org.apache.uima.cas.FeatureStructure)
    - org.apache.uima.jcas.cas.TOP
      - org.apache.uima.jcas.cas.AnnotationBase (implements org.apache.uima.cas.AnnotationBaseFS)
        - org.apache.uima.jcas.tcas.Annotation (implements org.apache.uima.cas.text.AnnotationFS)
          - edu.uchsc.ccp.uima.annotation.**CCPAnnotation**
            - edu.uchsc.ccp.uima.annotation.**CCPDocumentAnnotation**
              - edu.uchsc.ccp.uima.annotation.**CCPDocumentInformation**- edu.uchsc.ccp.uima.annotation.**CCPDocumentSection**- edu.uchsc.ccp.uima.annotation.**CCPDocumentSubSection**
              - edu.uchsc.ccp.uima.annotation.**CCPTextAnnotation**
                - edu.uchsc.ccp.uima.annotation.**CCPSemanticAnnotation**- edu.uchsc.ccp.uima.annotation.**CCPSyntacticAnnotation**
        - edu.uchsc.ccp.uima.annotation.**CCPAnnotationSet**- edu.uchsc.ccp.uima.annotation.**CCPAnnotator**- edu.uchsc.ccp.uima.annotation.**CCPSpan**
    - org.apache.uima.jcas.cas.TOP\_Type
      - org.apache.uima.jcas.cas.AnnotationBase\_Type
        - org.apache.uima.jcas.tcas.Annotation\_Type
          - edu.uchsc.ccp.uima.annotation.**CCPAnnotation\_Type**
            - edu.uchsc.ccp.uima.annotation.**CCPDocumentAnnotation\_Type**
              - edu.uchsc.ccp.uima.annotation.**CCPDocumentInformation\_Type**- edu.uchsc.ccp.uima.annotation.**CCPDocumentSection\_Type**- edu.uchsc.ccp.uima.annotation.**CCPDocumentSubSection\_Type**
              - edu.uchsc.ccp.uima.annotation.**CCPTextAnnotation\_Type**
                - edu.uchsc.ccp.uima.annotation.**CCPSemanticAnnotation\_Type**- edu.uchsc.ccp.uima.annotation.**CCPSyntacticAnnotation\_Type**
        - edu.uchsc.ccp.uima.annotation.**CCPAnnotationSet\_Type**- edu.uchsc.ccp.uima.annotation.**CCPAnnotator\_Type**- edu.uchsc.ccp.uima.annotation.**CCPSpan\_Type**

---


|  |  |  |
| --- | --- | --- |
| **Overview** \| **Package** \| Class \| **Tree** \| **Deprecated** \| **Index** \| **Help** | |  |
| **PREV**   **NEXT** | **FRAMES**    **NO FRAMES**     **All Classes** |  |


---
